# Time to improvement of pain, morning stiffness, fatigue, and disease activity in patients with ankylosing spondylitis treated with tofacitinib: a post hoc analysis

**DOI:** 10.1186/s13075-024-03313-w

**Published:** 2024-05-24

**Authors:** Victoria Navarro-Compán, Atul Deodhar, Rachid Bahiri, Andrew G. Bushmakin, Joseph C. Cappelleri, Jihane Rammaoui

**Affiliations:** 1 Rheumatology, University Hospital La Paz, IdiPAZ, Madrid, Spain; 2 Division of Arthritis and Rheumatic Diseases, Oregon Health & Science University, Portland, OR USA; 3 Department of Rheumatology, El Ayachi Hospital Medical University, Rabat, Morocco; 4 Statistical Research and Data Science Center, Pfizer Inc, Groton, CT USA; 5 Innovative Medicines, Emerging markets, AfME, Pfizer Inc, Casablanca, Morocco

**Keywords:** Ankylosing spondylitis, Spondyloarthritis, Tofacitinib, Pain, Fatigue, Disease activity, Patient-reported outcomes

## Abstract

**Background:**

Tofacitinib is an oral Janus kinase inhibitor for treatment of ankylosing spondylitis (AS). Time to improvement in core domains of AS was estimated in tofacitinib-treated patients with AS.

**Methods:**

This post hoc analysis used phase 3 trial data from patients with AS receiving tofacitinib 5 mg twice daily or placebo to week (W)16; all patients received open-label tofacitinib W16–48. Outcomes: nocturnal pain; total back pain; fatigue, spinal pain, peripheral joint pain/swelling, enthesitis, and morning stiffness (Bath AS Disease Activity Index [BASDAI] questions 1–6); BASDAI total score; AS Disease Activity Score (ASDAS). Median time to improvement events was estimated using non-parametric Kaplan-Meier models. Improvement events were defined as initial (first post-baseline observation) and continued (sustained for 2 consecutive visits) ≥ 30% and ≥ 50% improvement in back/nocturnal pain or BASDAI questions/total scores, or ASDAS improvement ≥ 1.1 and ≥ 2.0 points.

**Results:**

269 patients (tofacitinib: *n* = 133; placebo-to-tofacitinib: *n* = 136) were assessed. Median time to improvement was shorter, and more patients experienced improvements with tofacitinib vs. placebo-to-tofacitinib; differences observed from W2 (first post-baseline assessment). Median time to initial (continued) ≥ 30% pain improvement was 4 (4–8) weeks for tofacitinib vs. 24 (24) weeks for placebo-to-tofacitinib (8 [8] weeks post-switch). Median time to initial (continued) ≥ 50% improvement of pain, peripheral joint pain/swelling and enthesitis, morning stiffness, BASDAI total score, and fatigue was 8–24 (12–40) weeks with tofacitinib vs. 24–32 weeks (32 weeks–not estimable [NE]) with placebo-to-tofacitinib. Median time to initial (continued) ASDAS improvement ≥ 1.1 points was 4 (8) weeks for tofacitinib vs. 24 (24) weeks for placebo-to-tofacitinib, and NE for improvement ≥ 2.0 points with either treatment.

**Conclusions:**

Improvements in AS core domains occurred more rapidly with tofacitinib vs. placebo-to-tofacitinib. Half of tofacitinib-treated patients with AS will likely experience improvements ≥ 30% in pain and ≥ 1.1 points in ASDAS during month (M)1, ≥ 50% improvement in nocturnal pain and enthesitis by M2, and in morning stiffness by M3. Results show that initiating tofacitinib as soon as possible is associated with quicker improvements in AS core domains vs. delaying treatment.

**Trial registration:**

ClinicalTrials.gov, NCT03502616, 11 April 2018.

## Introduction

Ankylosing spondylitis (AS), also known as radiographic axial spondyloarthritis, is a chronic inflammatory disease characterized by a range of signs and symptoms, including back pain, morning stiffness, and fatigue [1, 2]. The global prevalence of AS varies from 6.5 to 540 per 100,000 persons, depending on geographical location [3]. AS can substantially impact patient well-being, productivity, and function, and decrease health-related quality of life [4, 5].

Achieving rapid and clinically meaningful improvement of AS symptoms is important for patients and physicians alike. The recommended treatment target in patients with AS is sustained remission or low disease activity, in addition to controlling symptoms, to maximize patients’ health-related quality of life [6–8]. Treatment guidelines for AS recommend using the AS Disease Activity Score with C-reactive protein (ASDAS) to assess disease activity, with achievement of ≥ 1.1 points defined as a clinically important improvement and ≥ 2.0 points defined as a major improvement [7, 9]. There are limited data on the timeframe for estimated improvements in pain and disease activity among patients with AS who initiate treatment. Insights into these timeframes would enable physicians, when deciding on treatment strategies, to inform their patients of when they might expect improvement of AS symptoms.

Tofacitinib is an oral Janus kinase (JAK) inhibitor for the treatment of AS. The efficacy and safety of tofacitinib 5 mg twice daily (BID) have been established in patients with active AS and an inadequate response or intolerance to ≥ 2 non-steroidal anti-inflammatory drugs in a 48-week phase 3, randomized controlled trial (RCT; NCT03502616) [10]. In the phase 3 RCT, tofacitinib treatment resulted in significant improvements vs. placebo in clinical measures pertaining to disease activity, back pain, fatigue, physical function, mobility, and health-related quality of life, with a significantly better clinical response vs. placebo occurring as early as week 2 (first post-baseline assessment) and sustained up to week 48 [10]. Moreover, a post hoc analysis of the phase 3 RCT showed that median time to initial improvement of 30% in fatigue (measured by the Functional Assessment of Chronic Illness Therapy-Fatigue [FACIT-F] total score) was 16 weeks in patients receiving tofacitinib 5 mg BID, whereas the median time for this event was not achieved up to week 16 in patients receiving placebo [11].

The aim of this post hoc analysis was to estimate the median time to initial and continued improvements in pain, peripheral joint pain/swelling and enthesitis, morning stiffness, fatigue, and disease activity in tofacitinib-treated patients with AS.

## Methods

### Data and patients

Data from a 48-week, phase 3, placebo-controlled RCT of tofacitinib in patients with active AS (NCT03502616) were included in this analysis. Full details have been reported previously [10].

Briefly, eligible patients aged ≥ 18 years diagnosed with AS who fulfilled the modified New York AS criteria (documented with central reading of the radiograph of the sacroiliac joints) [10, 12] were randomized 1:1 to receive tofacitinib 5 mg BID or placebo in the double-blind phase (weeks 0–16); thereafter, all patients received open-label tofacitinib 5 mg BID until week 48 [10].

### Assessments

The following outcomes were evaluated: total back pain and nocturnal pain (both measured using a numerical rating scale ranging from 0 [no pain] to 10 [most severe pain]); Bath AS Disease Activity Index (BASDAI); and ASDAS measured using C-reactive protein. These outcomes were assessed at baseline (day 1) and at weeks 2, 4, 8, 12, 16, 24, 32, 40, and 48.

A reduction in pain intensity of ≥ 30% was defined as “much improved” and a reduction in pain intensity of ≥ 50% was defined as “very much improved” per thresholds from The Initiative on Methods, Measurement, and Pain Assessment in Clinical Trials, which provides guidance for determining clinically important differences in Assessment in AS International Working Group-Outcome Measures in Rheumatology (ASAS-OMERACT) core domains, including pain intensity, in RCTs [1, 13].

The BASDAI is a validated instrument used to measure patient-reported disease activity in AS, comprising six questions (each answered on a numerical rating scale ranging from 0 [no disease activity] to 10 [high disease activity]) pertaining to the five major symptoms of AS: fatigue (question [Q]1), back/neck/hip pain (hereafter referred to as spinal pain [Q2]), peripheral joint pain/swelling (Q3), areas tender to touch (enthesitis; Q4), and intensity and duration of morning stiffness (Q5 and Q6) [14, 15].

The ASDAS is a composite score derived from three items of the BASDAI (spinal pain [BASDAI Q2], peripheral joint pain/swelling [BASDAI Q3], duration of morning stiffness [BASDAI Q6]), the Patient Global Assessment of Disease Activity, and C-reactive protein levels (mg/L) [14, 16]. Achievement of ASDAS ≥ 1.1 points is deemed to be a “clinically important improvement” while achievement of ASDAS ≥ 2.0 points is deemed to be a “major improvement” [9, 17].

### Statistical analyses

A series of time-to-event analyses were performed using non-parametric Kaplan-Meier models [18, 19]. The initial improvement event was defined as the time to the first post-baseline observation with an improvement of: ≥ 30% (“much improved”) or ≥ 50% (“very much improved”) in total back pain and nocturnal pain; ≥ 50% in BASDAI (total score and individual question scores) and morning stiffness (mean of BASDAI Q5 and Q6); and ≥ 1.1 (“clinically important improvement”) and ≥ 2.0 points (“major improvement”) in ASDAS. The continued improvement event was defined as the above criteria sustained for two consecutive visits.

Proportions of patients experiencing improvement events at each study visit were analyzed descriptively. For median times that were not achieved during the study, values were categorized as not estimable (NE). Analyses were performed using SAS® PROC LIFETEST.

## Results

### Patients

Data from 269 patients (133 randomized to tofacitinib 5 mg BID; 136 randomized to placebo-to-tofacitinib) were included in this post hoc analysis. Patient demographics, baseline disease characteristics, and baseline values for the instruments assessed in this analysis have been reported previously and were generally similar across treatment groups (mean [standard deviation] baseline values for the tofacitinib 5 mg BID and placebo-to-tofacitinib groups: total back pain, 6.9 [1.5] and 6.9 [1.6]; ASDAS, 3.8 [0.8] and 3.9 [0.8]; and BASDAI total score, 6.4 [1.5] and 6.5 [1.4]) [10].

### Median times to initial improvement events

Median time to initial improvement event of ≥ 30% (i.e., first post-baseline observation with an improvement of ≥ 30%) for both total back pain and nocturnal pain was 4 weeks for patients in the tofacitinib group, compared with 24 weeks (8 weeks since switch to tofacitinib) for the placebo-to-tofacitinib group (Table 1). Median times to initial improvement event of ≥ 50% (i.e. first post-baseline observation with an improvement of ≥ 50%) with tofacitinib were: 8 weeks for total back pain, nocturnal pain, spinal pain (BASDAI Q2), and enthesitis (BASDAI Q4); 10 weeks for peripheral joint pain/swelling (BASDAI Q3); 12 weeks for morning stiffness (BASDAI Q5 and Q6); and 24 weeks for fatigue (BASDAI Q1) (Table 1). Corresponding values for the placebo-to-tofacitinib group were: 24 weeks (8 weeks since switch to tofacitinib) for nocturnal pain, peripheral joint pain/swelling, and enthesitis; and 32 weeks (16 weeks since switching) for total back pain, fatigue, spinal pain, and morning stiffness (Table 1).

For ASDAS, median times to initial improvement events of ≥ 1.1 were 4 weeks with tofacitinib (NE to initial improvement events of ≥ 2.0 points). In the placebo-to-tofacitinib group, median times to initial improvement events of ≥ 1.1 were 24 weeks from baseline (8 weeks after switching from placebo to tofacitinib) and NE to initial improvement events of ≥ 2.0 points (Table 1). Median time to initial improvement event of ≥ 50% for BASDAI total score was 12 weeks for tofacitinib and 32 weeks (16 weeks after switching) for placebo-to-tofacitinib.

**Table 1:** Median time to initial improvement events (Kaplan–Meier analysis)

Improvement threshold	Median time, weeks (interquartile range)		*p* value
	Tofacitinib 5 mg BID (*N* = 133)	Placebo-to-tofacitinib^a^ (*N* = 136)	
≥ 30% improvement			
Total back pain^b^	4 (2–16)	24 (4–40)	< 0.0001
Nocturnal pain^b^	4 (2–24)	24 (8–32)	0.0003
≥ 50% improvement			
Total back pain^b^	8 (4–48)	32 (16–NE)	0.0001
Nocturnal pain^b^	8 (4–40)	24 (16–NE)	< 0.0001
BASDAI questions			
Fatigue (Q1)	24 (4–NE)	32 (12–NE)	0.0895
Spinal pain (Q2)	8 (4–NE)	32 (12–NE)	< 0.0001
Peripheral joint pain/swelling (Q3)	10 (2–NE)	24 (4–NE)	0.2070
Enthesitis (Q4)	8 (4–48)	24 (8–NE)	0.0250
Morning stiffness (Q5 and Q6)^c^	12 (4–NE)	32 (24–NE)	0.0091
BASDAI total score	12 (4–NE)	32 (16–NE)	0.0002
Improvement in ASDAS			
≥ 1.1 points	4 (2–20)	24 (12–40)	< 0.0001
≥ 2.0 points	NE	NE	0.0361

*p* values based on log–rank tests for differences in survival curves for tofacitinib vs. placebo-to-tofacitinib

^a^ Switched to open-label tofacitinib at week 16

^b^ Numerical rating scale (0–10)

^c^ Mean of BASDAI Q5 and Q6

*AS* ankylosing spondylitis, *ASDAS* Ankylosing Spondylitis Disease Activity Score with C-reactive protein, *BASDAI* Bath Ankylosing Spondylitis Disease Activity Index, *BID *twice daily, *NE* not estimable, *Q* question.

### Median times to continued improvement events

Median times to continued improvement events (i.e., initial improvement criteria sustained for 2 consecutive visits) of ≥ 30% with tofacitinib were 8 and 4 weeks for total back pain and nocturnal pain, respectively, compared with 24 weeks (8 weeks since switch to tofacitinib) for both outcomes for the placebo-to-tofacitinib group (Table 2). Median times to continued improvement events of ≥ 50% with tofacitinib were: 24 weeks for total back pain, spinal pain (BASDAI Q2), and morning stiffness (BASDAI Q5 and Q6); 12 weeks for nocturnal pain; 14 weeks for enthesitis (BASDAI Q4); 16 weeks for peripheral joint pain/swelling (BASDAI Q3); and 40 weeks for fatigue (BASDAI Q1) (Table 2). Corresponding values for the placebo-to-tofacitinib group were: ≥ 32 weeks (16 weeks since switch to tofacitinib) for nocturnal pain, peripheral joint pain/swelling, and enthesitis; and NE for total back pain and all other outcomes for BASDAI questions (Table 2).

For ASDAS, median times to continued improvement events of ≥ 1.1 and ≥ 2.0 points with tofacitinib were 8 weeks and NE, respectively; median time to continued improvement event of ≥ 50% for BASDAI total score was 24 weeks. Median times to continued improvement events of ≥ 1.1 and ≥ 2.0 points with placebo-to-tofacitinib were 24 weeks (8 weeks since switch to tofacitinib) and NE, respectively, whereas median time to continued improvement event of ≥ 50% for BASDAI total score was NE (Table 2).

**Table 2:** Median time to continued improvement events (Kaplan–Meier analysis)

Improvement threshold	Median time, weeks (interquartile range)		*p* value
	Tofacitinib 5 mg BID (*N* = 133)	Placebo-to-tofacitinib^a^ (*N* = 136)	
≥ 30% improvement			
Total back pain^b^	8 (2–32)	24 (16–NE)	< 0.0001
Nocturnal pain^b^	4 (2–40)	24 (16–NE)	< 0.0001
≥ 50% improvement			
Total back pain^b^	24 (4–NE)	NE	0.0003
Nocturnal pain^b^	12 (4–NE)	40 (24–NE)	< 0.0001
BASDAI questions			
Fatigue (Q1)	40 (8–NE)	NE	0.0212
Spinal pain (Q2)	24 (4–NE)	NE	< 0.0001
Peripheral joint pain/swelling (Q3)	16 (4–NE)	32 (12–NE)	0.0711
Enthesitis (Q4)	14 (4–NE)	40 (16–NE)	0.0017
Morning stiffness (Q5 and Q6)^c^	24 (4–NE)	NE	0.0037
BASDAI total score	24 (8–NE)	NE	0.0016
Improvement in ASDAS			
≥ 1.1 points	8 (2–NE)	24 (24–NE)	< 0.0001
≥ 2.0 points	NE	NE	0.2494

*p* values based on log–rank tests for differences in survival curves for tofacitinib vs. placebo-to-tofacitinib.

^a^ Switched to open-label tofacitinib at week 16

^b^ Numerical rating scale (0–10)

^c^ Mean of BASDAI Q5 and Q6

*AS* ankylosing spondylitis, *ASDAS* Ankylosing Spondylitis Disease Activity Score with C-reactive protein, *BASDAI* Bath Ankylosing Spondylitis Disease Activity Index, *BID *twice daily, *NE* not estimable, *Q* question.

### Proportions of patients experiencing improvement events at each study visit

**Fig. 1:**
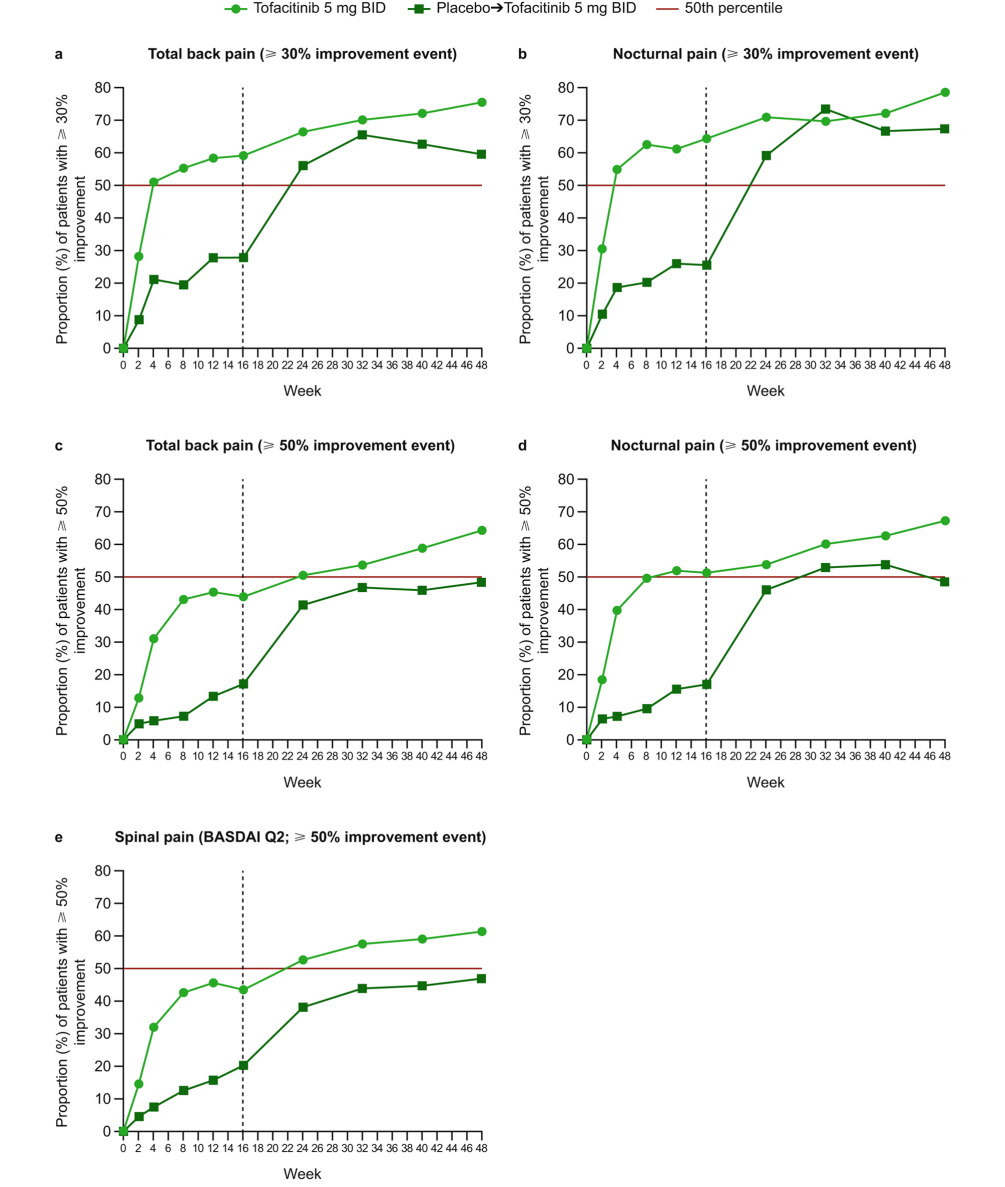
Improvement events in pain. Proportions of patients with ≥ 30% improvement in **a** total back pain and **b** nocturnal pain, and ≥ 50% improvement in **c** total back pain, **d** nocturnal pain, and **e** spinal pain (BASDAI Q2) at each study visit. Vertical dotted line indicates week 16, after which all patients received open-label tofacitinib until week 48. The 50th percentile line is to facilitate interpretation of the results. BASDAI, Bath Ankylosing Spondylitis Disease Activity Index; BID, twice daily; Q, question

**Fig. 2:**
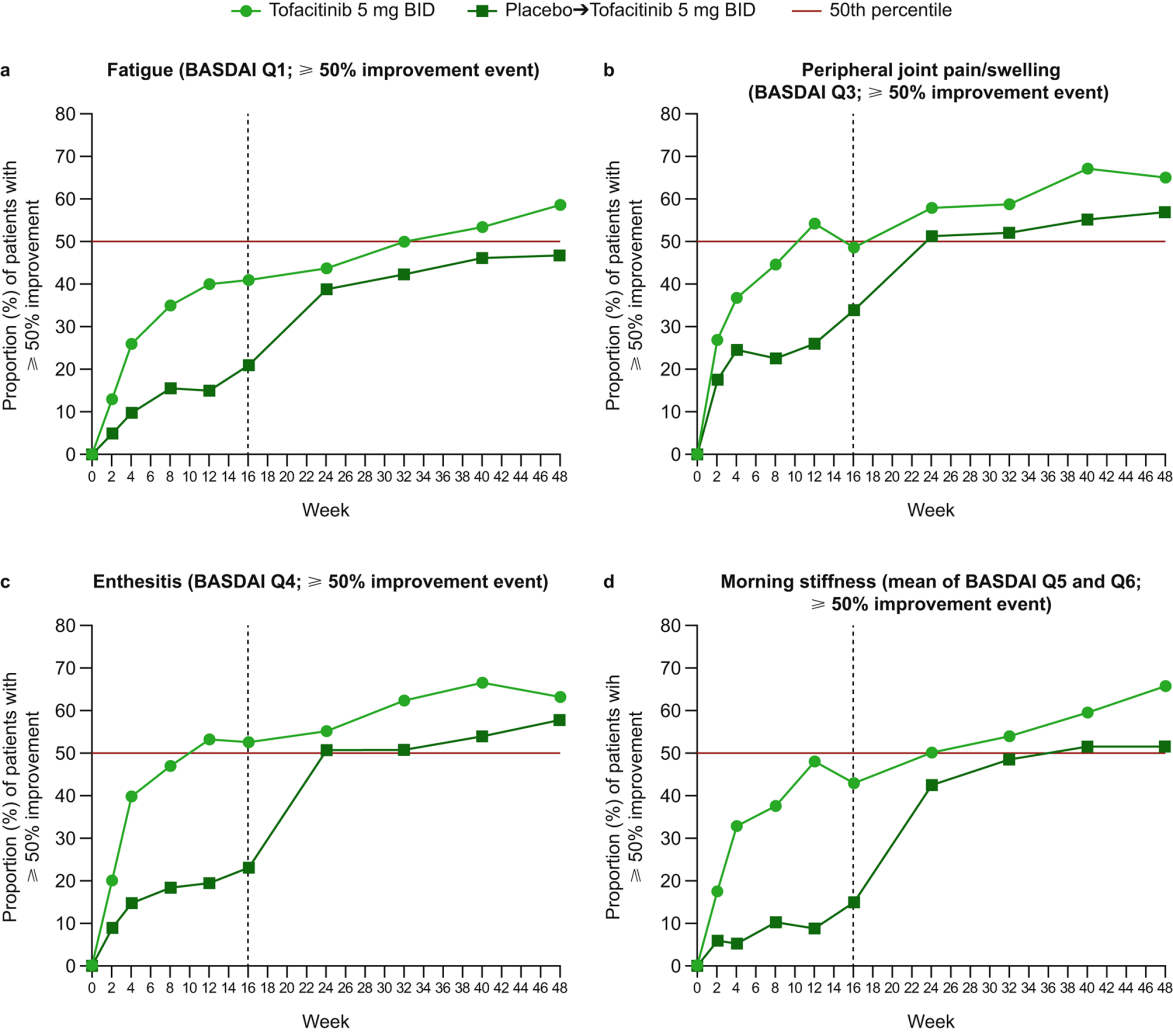
Improvement events in fatigue, peripheral outcomes, and morning stiffness. Proportions of patients with ≥ 50% improvement in **a** fatigue (BASDAI Q1), **b** peripheral joint pain/swelling (BASDAI Q3), **c** enthesitis (BASDAI Q4), and **d** morning stiffness (mean of BASDAI Q5 and Q6) at each study visit. Vertical dotted line indicates week 16, after which all patients received open-label tofacitinib until week 48. The 50th percentile line is to facilitate interpretation of the results. BASDAI, Bath Ankylosing Spondylitis Disease Activity Index; BID, twice daily; Q, question

**Fig. 3:**
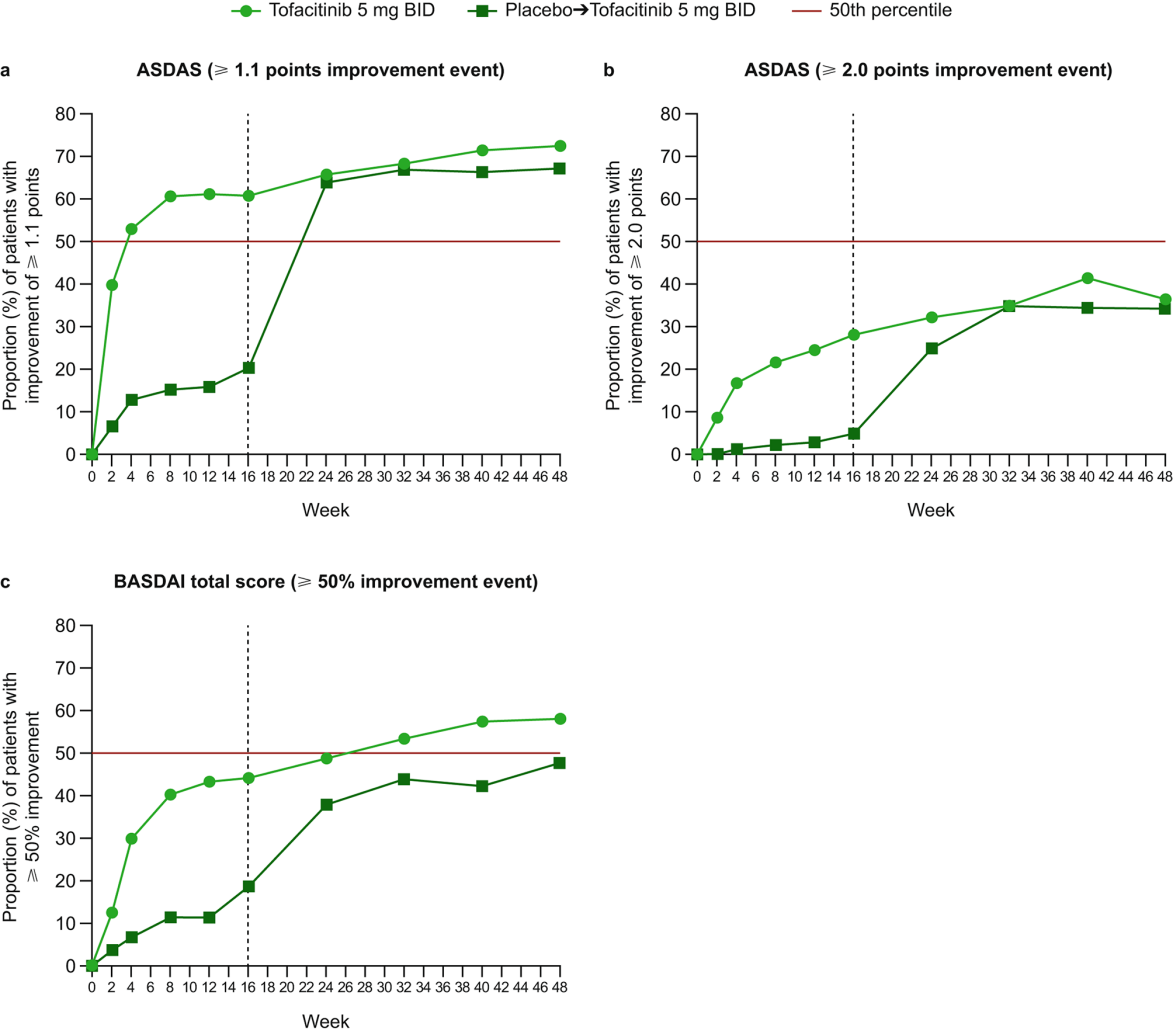
Improvements in ASDAS and BASDAI total score. Proportions of patients with improvement in ASDAS of **a** ≥ 1.1 points and **b** ≥ 2.0 points, and **c** ≥ 50% in BASDAI total score at each study visit. Vertical dotted line indicates week 16, after which all patients received open-label tofacitinib until week 48. The 50th percentile line is to facilitate interpretation of the results. ASDAS, Ankylosing Spondylitis Disease Activity Score C-reactive protein; BASDAI, Bath Ankylosing Spondylitis Disease Activity Index; BID, twice daily

Generally, at most study visits, greater proportions of patients receiving tofacitinib experienced improvements in pain, morning stiffness, fatigue, and disease activity, compared with patients in the placebo-to-tofacitinib group, with the difference in response compared with placebo-to-tofacitinib observed from week 2. Improvements with tofacitinib were sustained to week 48 (Figs. 1, 2 and 3). After switching from placebo to open-label tofacitinib at week 16, the proportions of patients with improvements in pain, morning stiffness, fatigue, and disease activity approached those observed in the tofacitinib group within 8 weeks of switching, but generally remained lower than those for the patients who received tofacitinib throughout the study (Figs. 1, 2 and 3).

For nocturnal pain, 31% and 18% of patients experienced improvement events of ≥ 30% and ≥ 50%, respectively, at week 2 with tofacitinib (placebo-to-tofacitinib: 11% and 7%) (Fig. 1b, d). At week 4, over half of patients experienced an improvement event of ≥ 30% in both total back pain and nocturnal pain with tofacitinib (placebo-to-tofacitinib: 21% and 19%), with the proportions continuing to increase to week 48 (Fig. 1a, b). At the end of the double-blind period at week 16, improvements of ≥ 30% in total back pain and nocturnal pain were experienced by 59% and 64% of patients in the tofacitinib group, respectively (placebo-to-tofacitinib: 28% and 26%, respectively; Fig. 1a, b), while improvements of ≥ 50% in total back pain, nocturnal pain, and spinal pain (BASDAI Q2) were experienced by 43–51%, and 17–20%, of patients in the tofacitinib and placebo-tofacitinib groups, respectively (Fig. 1c, d, e). By week 24, at least half of patients in the tofacitinib group had experienced an improvement event of ≥ 50% in total back pain, nocturnal pain, and spinal pain, whereas in the placebo-to-tofacitinib (8 weeks since switch to tofacitinib) group, 42%, 46%, and 38% of patients had experienced an improvement event of ≥ 50% in these symptoms, respectively (Fig. 1c, d, e).

At week 2, the proportions of patients with improvement events of ≥ 50% in fatigue (BASDAI Q1), peripheral joint pain/swelling (BASDAI Q3), enthesitis (BASDAI Q4), and morning stiffness (mean of BASDAI Q5 and Q6) ranged from 13 to 27% with tofacitinib (placebo-to-tofacitinib: 5–18%) (Fig. 2). At week 16, the proportions of patients with an improvement event of ≥ 50% in peripheral joint pain/swelling, enthesitis, morning stiffness, and fatigue with tofacitinib ranged from 41–52% (placebo-to-tofacitinib: 15–34%) (Fig. 2). By week 24, at least half of patients in both the tofacitinib and placebo-to-tofacitinib treatment groups (8 weeks after switching) had experienced an improvement event of ≥ 50% in peripheral joint pain/swelling (BASDAI Q3) and enthesitis (BASDAI Q4) (Fig. 2b, c). For morning stiffness (mean of BASDAI Q5 and Q6), half of patients in the tofacitinib group experienced an improvement of ≥ 50% by week 24, while approximately half of the patients in the placebo-to-tofacitinib group experienced an improvement of ≥ 50% by week 40 (Fig. 2d). For fatigue (BASDAI Q1), half of patients receiving tofacitinib had experienced an improvement event of ≥ 50% by week 32, while less than half of patients who switched from placebo improved by ≥ 50% at any study visit (Fig. 2a).

For ASDAS, the proportions of patients who experienced improvement events of ≥ 1.1 points and ≥ 2.0 points at week 2 were 40% and 8%, respectively, for tofacitinib (placebo-to-tofacitinib: 7% and 0%) (Fig. 3a, b). Over half of patients experienced an improvement event of ≥ 1.1 points by week 4 in the tofacitinib group, compared with by week 24 (8 weeks after switching) for the placebo-to-tofacitinib group. At week 16, the proportion of patients with an improvement event of ≥ 1.1 points was 61% with tofacitinib (placebo-to-tofacitinib: 20%) (Fig. 3a). For both treatment arms, less than half of patients experienced an improvement event of ≥ 2.0 points at any study visit (Fig. 3b). Improvement events of ≥ 50% for BASDAI total score were experienced by 12% and 4% of patients receiving tofacitinib and placebo-to-tofacitinib, respectively, at week 2 (Fig. 3c). Over half of patients in the tofacitinib group experienced an improvement event of ≥ 50% for BASDAI total score at week 32. Less than half of patients in the placebo-to-tofacitinib group experienced an improvement event of ≥ 50% at any study visit, although the proportion with improvement events increased from 19% at week 16 (tofacitinib: 44%) to 38% at week 24 (8 weeks after switching to tofacitinib) (Fig. 3c).

## Discussion

Patients with AS and their physicians want to know the answers to the key questions of when the patient can expect to start feeling better and when their treatment is likely to reach its maximum effect. Insights into these aspects of therapy would be helpful when choosing treatment strategies and managing patient expectations. However, data on timeframes for estimated improvements in pain, morning stiffness, fatigue, and disease activity among patients with AS initiating treatment are limited. In this post hoc analysis, we attempted to answer these key questions in patients with AS receiving tofacitinib, by investigating the time to meaningful improvements in pain, morning stiffness, fatigue, and disease activity measures, which are important treatment goals for patients and physicians [8, 20].

In this post hoc analysis of data from a phase 3 RCT of patients with AS, median times to initial (first post-baseline observation)/continued (sustained for two consecutive visits) improvement events in total back pain, nocturnal pain, fatigue, spinal pain, peripheral joint pain/swelling, enthesitis, morning stiffness, ASDAS and BASDAI total score were generally shorter for patients initially randomized to tofacitinib 5 mg BID during the double-blind part of the study vs. switched from placebo to open-label tofacitinib 5 mg BID at week 16. For most study visits, more patients experienced improvement events with tofacitinib 5 mg BID vs. placebo-to-tofacitinib, with differences between treatment arms observed as early as week 2 (first post-baseline assessment). Future studies may benefit from collecting and analyzing data in the days immediately following initiation of treatment with tofacitinib.

Improvements with tofacitinib 5 mg BID were generally sustained to week 48. Our findings suggest that, after initiating tofacitinib 5 mg BID, half of patients could experience an initial ≥ 30% improvement in total back pain and nocturnal pain (“much improved”) by week 4 (weeks 4–8 for continued improvement). Additionally, by weeks 8–12 of tofacitinib 5 mg BID treatment, half of patients could experience ≥ 50% improvement in pain (“very much improved”, total back pain, nocturnal pain, and spinal pain), peripheral symptoms (peripheral joint pain/swelling and enthesitis), and morning stiffness (weeks 12–24 for continued improvement). A “clinically important improvement” in ASDAS (≥ 1.1 points) was observed in half of the patients by week 4 (week 8 for continued improvement), and a ≥ 50% improvement in BASDAI total score was observed in half of the patients by week 12 (week 24 for continued improvement). The shorter time to improvement for ASDAS compared with BASDAI total score may indicate that ASDAS provides a more sensitive measure of change in symptoms.

As discussed above, median times to initial/continued improvement events were generally longer with placebo-to-tofacitinib vs. tofacitinib 5 mg BID. For example, for patients originally randomized to placebo who switched to active treatment with open-label tofacitinib 5 mg BID, median time to initial ≥ 30% improvement in total back pain and nocturnal pain was 24 weeks, i.e., 8 weeks after starting active treatment. In contrast, median time to initial ≥ 30% improvement in total back pain and nocturnal pain for patients who were originally randomized to tofacitinib 5 mg BID was only 4 weeks. The response to tofacitinib after switching from placebo may have been influenced by the open-label nature of this part of the study.

Symptoms corresponding to pain were among the first to show improvement, with improvements for morning stiffness and fatigue occurring later. A previous post hoc analysis of the phase 3 RCT showed that median time to initial improvement of 30% in fatigue (FACIT-F total score) was 16 weeks in patients receiving tofacitinib 5 mg BID [11]. In the tofacitinib 5 mg BID group, median time to both initial and continued improvement of ≥ 30% in nocturnal pain was 4 weeks; this suggests that half of patients could expect to experience sustained improvements in nocturnal pain within the first month of initiating treatment. Median times to initial/continued improvements in pain outcomes, peripheral joint pain/swelling, and enthesitis were generally shorter (4–10 weeks/4–24 weeks) than median times to initial/continued improvements in fatigue, morning stiffness, and BASDAI total score (12–24 weeks/24–40 weeks). This difference in time to improvement in domains might be accounted for by tofacitinib’s mechanism of action, as well as the multifactorial etiology of some domains. For example, a recent study demonstrated that improvement in fatigue is mediated by the combined effects of tofacitinib treatment on morning stiffness and pain, providing an explanation for why improvements may occur more rapidly in some domains vs. others [21].

A recent post hoc analysis of the tofacitinib phase 3 RCT in patients with AS showed that greater proportions of patients achieved from baseline ≥ minimum clinically important difference at week 16 with tofacitinib 5 mg BID vs. placebo for pain (total back pain, BASDAI overall spinal pain, and nocturnal spinal pain) (defined as a decrease ≥ 1), and fatigue (FACIT-F total [defined as an increase ≥ 4] and BASDAI fatigue [defined as a decrease ≥ 1] scores) [22]. Our data complement these findings, suggesting that half of patients initiating tofacitinib 5 mg BID at baseline could expect to experience an initial ≥ 50% improvement in nocturnal pain, total back pain, and spinal pain by 8 weeks and fatigue by 24 weeks.

Our findings on time to improvement in pain with tofacitinib in patients with AS are consistent with data from a post hoc analysis using data from two phase 3 RCTs of patients with psoriatic arthritis treated with tofacitinib 5 mg BID [23]. In patients with psoriatic arthritis, improvements of ≥ 30% and ≥ 50% in pain were also experienced more rapidly and by more patients with tofacitinib, compared with placebo [23]. Moreover, the current findings align with those of a recent post hoc analysis of three RCTs in patients with AS and psoriatic arthritis receiving the JAK inhibitor upadacitinib, which demonstrated median times to ≥ 30% and ≥ 50% improvements in pain of 4.1 and 8.6 weeks, respectively [24]. Also in alignment with the current study, a post hoc analysis of two RCTs showed that the time to response in patients with psoriatic arthritis was shorter for those treated with tofacitinib 5 mg BID and adalimumab 40 mg once every 2 weeks than for those who, at month 3, switched from placebo to tofacitinib 5 mg BID, for outcomes such as the Health Assessment Questionnaire-Disability Index (approximately 1 month for tofacitinib and adalimumab compared with approximately 4 months for placebo-to-tofacitinib). In addition, patients initially treated with tofacitinib or adalimumab were more likely to have a minimal disease activity composite score response within the first 3 months compared with patients who switched from placebo to tofacitinib at month 3 [25].

Limitations of this study include the post hoc nature of the analysis, the lack of an active treatment comparator in the RCT, and the fact that the RCT was not designed to compare time-to-event outcomes, as patients were assessed according to a protocol-determined fixed schedule of regular clinic visits. Generalizability of the findings for patients with milder disease is limited because the primary trial population included patients with high baseline disease activity. Furthermore, the study design only incorporated double-blind comparisons with placebo up to week 16; thereafter, all patients received open-label tofacitinib. In the open-label phase, patient awareness of the treatment that they received may have impacted their expectations and influenced median time to improvement.

## Conclusions

In patients with active AS, initial/continued improvements in pain, morning stiffness, fatigue, and disease activity measures, which are of importance to patients and physicians [8, 20], were experienced more rapidly, and by more patients, in those receiving tofacitinib 5 mg BID vs. those receiving placebo then switching to tofacitinib at week 16. The shortest time to improvements was observed for pain, ASDAS, and enthesitis, followed by peripheral joint pain/swelling, morning stiffness, and BASDAI, with fatigue improving later. During the first month of tofacitinib treatment, it is expected that half of patients will experience ≥ 30% improvement (“much improved”) in total back pain and nocturnal pain, and a clinically important improvement of ≥ 1.1 points in ASDAS. Half of patients may also expect to experience a ≥ 50% improvement (“very much improved”) in nocturnal pain and enthesitis during the first 2 months, and a ≥ 50% improvement in morning stiffness during the first 3 months of tofacitinib treatment. Improvements with tofacitinib 5 mg BID were sustained up to week 48. For patients with persistently high disease activity despite conventional treatments, initiating tofacitinib as soon as possible was associated with quicker improvements in core domains of AS vs. delaying treatment.

## Data Availability

Upon request, and subject to review, Pfizer will provide the data that support the findings of this study. Subject to certain criteria, conditions, and exceptions, Pfizer may also provide access to the related individual de-identified participant data. See https://www.pfizer.com/science/clinical-trials/trial-data-and-results for more information.

## Abbreviations

AS: Ankylosing spondylitis
ASAS-OMERACT: Assessment in AS International Working Group-Outcome Measures in Rheumatology
ASDAS: Ankylosing Spondylitis Disease Activity Score
BASDAI: Bath Ankylosing Spondylitis Disease Activity Index
BID: Twice daily
FACIT-F: Functional Assessment of Chronic Illness Therapy-Fatigue
JAK: Janus kinase
M: Month
NE: Not estimable
Q: Question
RCT: Randomized controlled trial
W: Week
